# Western Anthelmintics in Early Twentieth‐Century China Colonial Practices and Knowledge on “Tropical Diseases” of the In/between**

**DOI:** 10.1002/bewi.202400001

**Published:** 2024-11-02

**Authors:** Dominik Merdes

**Affiliations:** ^1^ Chair of the History of Knowledge of Modern Societies Helmut Schmidt University Hamburg; ^2^ Department of the History of Science and Pharmacy Technical University of Braunschweig Braunschweig

**Keywords:** drug history, coloniality, *Fasciolopsis buski*, hookworm, roundworm, santonin, thymol, China, Medical Missionary Association, knowledge generating structures

## Abstract

Protestant (medical) missionaries were the main proponents of Western medicine in China after the Opium Wars. Several studies have highlighted how they used spectacular surgery as a means of gaining public trust. As well as surgery, they also administered anthelmintic drugs such as santonin as a tool of persuasion and conversion. Many anthelmintic drugs of the European *materia medica* had a colonial history. My paper analyses how *coloniality* materialised in medical practice and anthelmintics in China. For the late nineteenth century, I will examine the colonial practices in which the drug santonin was involved. At the time, santonin was the drug of choice for treating roundworm. In the early twentieth century, medical missionaries became involved in parasitological research on parasitic worms such as hookworm and *Fasciolopsis buski*. For this period, I will explore how new knowledge about anthelmintics emerged in the scattered knowledge space of China, and how it related to colonialism and imperialism.

Western *materia medica* is deeply intertwined with colonialism. This is true of the plant‐based *materia medica* that dominated for centuries, as well as the industrial synthetic drugs prevailing since the turn of the century. Many herbal medicines were appropriated by Europeans in colonised land. The co‐constitution of *materia medica* and colonialism is evident at least for the period since the early sixteenth century, when drugs appropriated in the Americas flew to Europe.^1^ Drugs became involved in an exploitative structure that Aníbal Quijano and others call “coloniality.”^2^ The production of alkaloids, chemotherapeuticals and other supposedly “Western” medical achievements depended heavily on *coloniality*, which continues to this day.

One group of drugs that may demonstrate the *coloniality* of drugs are the anthelmintics or vermifuges. Anthelmintics are used to expel worms. In combination with other drugs, especially purgatives, they have been used for centuries against worms visible to the naked eye, such as roundworms or tapeworms. Since the late nineteenth century, anthelmintics have also been used against much smaller worms that can only be detected under a microscope, such as hookworm or bilharzia.^3^

Anthelmintics were also used in China. Compared to other regions of colonisation, the penetration of Western imperialism into China was relatively late. The example of China also shows that imperialism and colonialism cannot be reduced to the decisions of particular states or politicians. While they were instrumental in establishing these exploitative structures, they were only parts of a wider imperial assemblage that also included, for example, social desires or non‐state actors such as missionaries.^4^ After China was forced to “open up” for Western imperialism following the Opium Wars (1839–1842 and 1856–1860), it was mainly medical missionaries, Western customs surgeons (formally employed by the Chinese government) and various kinds of colonial doctors who used Western anthelmintics in China. Because of Patrick Manson's research on filarial worms, southern China became an important element in the history of helminthology, parasitology and so‐called tropical medicine.^5^ As a medical officer of the Chinese Imperial Maritime Customs Service, Manson demonstrated that filaria, which he considered to be the cause of diseases such as elephantiasis, were transmitted by mosquito bites in the 1870s. The life history of the worm became the blueprint for Ronald Ross's malaria research in British India, which provided the foundational knowledge for understanding the malaria transmission cycle.^6^ As for anthelmintics, there were no such landmarks in China. However, it is worth exploring the history of Western anthelmintics in China. On the one hand, there was knowledge produced in missionary hospitals or in industrial settings such as coal mines that caused less of a stir. On the other hand, we can learn about colonial drug practices and the interactions with Chinese societies that left their mark on “Western” drugs. After Manson, it were especially medical missionaries that played an important role in parasitological research in China.^7^ They were rather unusual agents in the field of tropical medicine. The discourse of tropical medicine, which materialised in publications in scientific journals and discussions on international conferences, was dominated by colonial doctors and parasitology experts from Europe and the US. However, while the main aim of the missionary societies was to convert the Chinese to Christianity, and their scientific activities were viewed critically by “secular” parasitologists, they became involved where other practitioners of Western medicine were rare. In the following, I will analyse how colonialism materialised in anthelmintics in/between^8^ medical missionaries and Chinese societies in the late nineteenth and early twentieth centuries. Publications written by Western physicians and missionaries form the source base. Did anthelmintics take on the role of a “tool of empire,” as Daniel Headrick claims for quinine?^9^ And how did the interactions between medical missionaries and local populations affect the materialisation of drugs? The first part of this paper examines the colonial practices in which the drug santonin was involved. Santonin was a substance isolated from so‐called worm seeds. In the nineteenth century it became the drug of choice for treating roundworm infections. In the second part I explore how new knowledge about anthelmintic drugs emerged in southern China. Drawing on concepts from historical epistemology, I will try to understand how this was possible in the scattered knowledge space of China, and how this knowledge was linked to imperial missions. The final part summarises the findings and discusses how they relate to *coloniality*.

## 1. Santonin as Colonial Miracle and Mechanistic Wonder Weapon

After the Opium Wars, missionaries, medical missionaries that had studied medicine but also laymen who practised medicine, became the main proponents of Western medicine in China. Several accounts of the history of Western medicine in China claim that (medical) missionaries skilfully staged spectacular medical treatments such as tumour or cataract surgery as miracles. In this way, medicine was used as a tool of conversion. Li Shang‐Jen points out that the medical miracles initially selected particular cases to stage medical miracles. In order to highlight the effects of Western medicine as impressively as possible, patients with a bad prognosis were often not treated at all.^10^ Drugs also functioned as such tools of conversion, although they may have been less spectacular tools. Medical missionaries used quinine, iodine ointment, opium pills and sulphur ointment (for scabies), among other things.^11^ One group of diseases that were mainly treated with drugs were worm infections. Especially the roundworm or *Ascaris lumbricoides* was frequently found in China (the reports often do not mention the biological species, but simply refer to “roundworms”). In 1866, the medical missionary James Laidlaw Maxwell (1836–1921) of the Presbyterian Church of England reported on the prevalence of roundworms in Taiwan: “*Lumbrici* are so common that the people here do not take any account of them until associated with some other complaint more urgent in its symptoms.”^12^ Like others, he pictured China as worm‐infested area. Roundworms could be effectively expelled with santonin, a white powder isolated from worm seeds.

When santonin entered China, it was already linked to a long colonial history. The medicinal worm seeds, also called *Semen cinae* or *Semen santonici*, from which santonin was extracted, had been used by European doctors for centuries as an anthelmintic. However, the parent plant had long escaped the control of Western medicine and botany. It was not until the late 19th nineteenth century that the plant was more clearly identified. At that time, the plant *Artemisia cina*, from which santonin was extracted, was taken from the Syrdarya region of Turkestan, which was then controlled by the Russian Empire. Botanically, the aromatic, bitter‐tasting worm seeds were not seeds, but small, closed flower heads of various *Artemisia* species. Unlike worm seeds, santonin was tasteless and odourless. Santonin crystals were first observed in quick succession in 1830 by Apothecary Kahler in Düsseldorf and Joachim August Alms (1803–1847) in Penzlin.^13^ The name santonin, alluding to the plant name *Santonicum* and the *Semen santonici*, was established a little later.^14^

Even though santonin quickly became a successful industrial product, the appropriation of the required drug was rather uncontrolled. In Europe, the corresponding worm seeds were bought from traders and used medicinally before the parent plant had been botanically defined. Although it was assumed to be a species of *Artemisia*, it was difficult to narrow it down botanically and historically. In the second half of the nineteenth century, there was still no consensus on the plant from which the drug was derived. Even after the isolation of the presumed active principle, the drug was defined less botanically than by the trade routes it took.^15^ On the one hand, the worm seeds were defined by their origin from the Central Steppe, on the other hand by their association with the Arab trade. Some of the names that circulated for worm seeds, such as *Semen cinae halepense* or “Levant wormseed” were geographically coloured. While these names suggest Aleppo and the Levant, the “cinae” in *Semen cinae* was also read by some authors as a reference to China.^16^ Later, the Swiss pharmacist and chemist Friedrich August Flückiger (1828–1894) suggested that the name *Semen cinae* was derived from the Italian diminutive for seed, “Semenzina.”^17^

The unclear origin of the worm seeds had no effect on the spread of santonin. Heinrich Emanuel Merck (1794–1855) isolated santonin from worm seeds in Darmstadt as early as 1833. After Merck, Gehe & Co. started producing santonin in 1876, followed later by Johann Diedrich Bieber, C. F. Böhringer & Söhne, T. and H. Smith, and H. Mauer & Co.^18^ In the USA, santonin was one of the first products of Pfizer, founded in 1849. German manufacturers tried to move santonin production to the *Artemisia cina* growing areas in Syrdarya, Turkestan. In 1883, a santonin factory was built in Orenburg by H. Mauer & Co.^19^ With the help of German engineers, another factory was built in the city of Shymkent by the Russian company Iwanoff & Ssawinkoff.^20^ Conquered by Russia in 1864, Shymkent was located directly in Syrdarya. In the late nineteenth century, Russia dominated the santonin market. Santonin production clashed with the interests of the nomads of Syrdarya who, according to Gerhard Madaus, used the plant as fuel.^21^

In late nineteenth‐century China, santonin was used by both medical missionaries and lay people. One layman who administered santonin was James Gilmour (1843–1891), an evangelical missionary working for the London Missionary Society. In 1889, he reported on the occurrence of worm infections in Mongolia, which caused a clinical picture unknown to him.^22^ His report appeared in the *China Medical Missionary Journal*, which had been founded in 1887 to facilitate exchange between missionary doctors scattered over much of China. At times, Gilmour lived in a tent, which served as his chapel and dispensary.^23^ He sold medicines at markets, where he took the opportunity to wrap them in packages printed with Bible verses.^24^ The fact that Gilmour, as an evangelical missionary, provided medical care to people was not unusual at the time.

Like many other Europeans, Gilmour found the living conditions in the Qing Empire extremely dirty and unhygienic. He directly associated the dirt on the streets with worms living inside the human body:

One thing that has amazed me is the extent to which worms are present in the stomach and intestines of the inhabitants of eastern agricultural Mongolia. The Chinese do not use water‐closets; pigs act as scavengers, but seem to refuse worms; and a foreigner living among the natives is struck by the quantity of worms passed in the ordinary course of nature.^25^

Gilmour claimed that worm infections were very common. He correlated this with the use of anthelmintics: “Looking around I am amazed to find how extensive is the trade in anthelmintic bon‐bons. Every grocer's shop seems to have them, and nearly every peddler, selling odds and ends throughout the country, has a bottle or two in his store.”^26^

Gilmour himself, who did not distinguish between different worm species, used santonin to treat worm disease. Due to santonin's ability to expel large numbers of worms when given in small doses, the drug proved useful in his missionary efforts. The drug allowed him to make contact with the people. Although worm remedies were common among the locals, Gilmour's story depicts the worm removal with santonin almost as a miracle: “Santonine works wonders. The Chinese here are in the habit of saying that everyone has worms, but they are surprised at the large results of small doses of medicine.”^27^ According to this statement, santonin was primarily a miracle in the eyes of the locals. The healer from the advanced, scientific world brings light to the backward, superstitious Chinese. Gilmour established kind of a colonial arrangement of the miracle, which comes close to the staging of operations in mission hospitals. Similar to certain surgical interventions, santonin operated as a miracle cure that worked across epistemic divides, astonishing Western doctors as well as the cured.^28^ Nevertheless, there was an asymmetry in the perception of the Western healers. While the physicians believed that they were using remedies rationally as technical means and controlling their effects, the healed were seen as emotional beings who experienced healing in the irrational sphere of the magical. Despite the colonial role allocation, Gilmour himself seems to have placed his hopes in santonin as a miracle cure. He thought it was quite possible that worm diseases were a kind of universal ailment in China. Half‐jokingly, he prophesied:

Meantime, till more information is forthcoming on this subject, when doctors tell me laughingly that it is my ignorance of other diseases which leads me to give undue prominence to this, I retort, in the same good humour, that their learning leads them astray and sets them to treating for indigestion and heart disease patients who could be set all right by a couple of Santonine lozenges.^29^

Finally, Gilmour asked the readers of the *China Medical Missionary Journal* to gather more information about worm diseases.^30^

Santonin was also the standard treatment for roundworms in many missionary hospitals in China. Although Chinese *materia medica* knew of several remedies for roundworms or Huichong ( 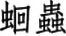), santonin soon became popular with the Chinese people. The medical missionary W. M. Riddel, who proselytised for the Presbyterian Church of England in the Hakka region, reported in 1891 on the appropriation of Western medicines by the local population. Santonin was among the medicines mentioned:

The Chinese are quick to find out what they can use, and have of late come to know the names and uses of not a few of our medicines. Best known perhaps is Quinine. […] They have come to know the various uses of iodine, santonin for worms, benzoated zinc ointment for eczema, zinc lotion for the eye, sulphur ointment for scabies. They are beginning to know ipecac. and cod‐liver oil.^31^

Whereas surgery was tied to the location of the hospital, drug therapy was able to spread. But in the in/betweens of the environment of the missionary hospitals it often spread in unforeseen ways. For example, the modalities of administration changed in the interstice between the doctor and the patients. The observation that patients varied the dosage of the drugs mentioned and made their own diagnoses led Riddel to fear a loss of authority: “They sometimes carry their knowledge too far. They believe in medicine and ignore the physician.”^32^

While the use of santonin was mentioned only in passing in medical publications of the second half of the nineteenth century, it appeared increasingly in articles from the turn of the century onwards. However, these developments were less a response to Gilmour's call for research into worm disease than to the wider discourse of tropical medicine. In the early years of the twentieth century, several medical missionaries arrived in China who had acquired some knowledge of parasitology during their studies. For instance, John Preston Maxwell (1871–1961) of the Presbyterian Church of England, a son of James Laidlaw Maxwell, studied worms soon after his arrival in Fujian. In a paper published in the *Journal of Tropical Medicine* in 1900, he published his findings that roundworms are transmitted via eating raw garlic and leeks, on which he found roundworm eggs and embryos, in the Zhangpu village.^33^ The medical missionary also went into more detail about the treatment of worms with santonin. He answered the question of how to treat a roundworm infection succinctly: “Give santonin.”^34^ Maxwell administered worm cures with santonin not only to treat patients. He also used it to see if certain people would expel roundworms after a dose of santonin. In addition to the life history and therapy of the worm, he was also interested in its epidemiology. In boarding schools in Zhangpu, he used santonin to determine the level of roundworm infection among the pupils.^35^

Maxwell also treated patients suffering from symptoms of worm disease. Like leading parasitologists, Maxwell stressed the importance of informing patients about the side effects of santonin. These included yellow‐coloured vision, colouration of the urine, vomiting and fever attacks. He felt it was particularly important to pass this information on to “a people like the Chinese.”^36^ The information given to patients about the side effects and the use of the drug in healthy people suggest that Maxwell was not staging santonin as a colonial miracle cure. Rather, he was staging the miracles of rational therapy and using the drug as a tool for the production of medical knowledge. By focusing on research, the health of the patient became secondary. Notwithstanding the rationality of the treatment, the effects of santonin seem to have overwhelmed the professional observer himself: “A dose of castor oi [sic!] and santonin caused the passage of a large number of worms and the vomiting of two or three, and the discomfort disappeared as if by magic.”^37^ Despite his efforts to obtain accurate scientific data, his therapeutic efforts seem to have been driven by a desire for specific miracle cures.

Other medical missionaries observed an overly generous use of santonin among their fellow missionaries, which they denounced as dangerous. One of the first to point out the problem was the American medical missionary Oliver Tracy Logan (1870–1919), perhaps because he had experienced the side effects of santonin first‐hand. After learning from a colleague that it was common practice among missionaries in China to administer santonin as a monthly worming treatment, he had a bad experience with the drug:

Acting upon his advice I tried to do my duty, but the result was that I made myself one of the most miserable of beings for many hours, during which time the world looked literally and figuratively of an icteroid hue, without, however, increasing the mortality of the interesting lumbricoid.^38^

Nevertheless, Logan also used the product liberally at first.^39^ During his short time in China, Logan then observed a change in the use of santonin in the wider missionary medical structure, which he attributed to the microscope: “This was in the former days of our mission. Now we insist upon seeing traces of game at least, before we fire at ascaris with our therapeutic gun, except in dispensary practice where this is not practicable.”^40^ The “traces of game” were the eggs of the worm *Ascaris lumbricoides*, which could be seen under the microscope. The microscope was also used to detect bacteria, protists such as plasmodia, the malaria parasite, and pathological changes in tissues. Detecting worm eggs was a relatively simple procedure, as they were quite large and did not require staining to be seen.

Preston Maxwell had also stressed the diagnostic importance of the microscope in 1900. Ultimately, however, for him symptoms were equally reliable criteria.^41^ Logan's tone became sharper. When it came to malaria and quinine treatment, it was a matter of life and death for him: “Now in the writer's opinion any physician, whose Board of missions could afford to furnish him a microscope, who treats a case of continued fever without positive evidence of malaria, with such doses of *quinine*, is culpable in the extreme.”^42^

The violent metaphors Logan uses are also notable. The self‐portrayal as a parasite hunter became common among medical missionaries in China, and the reference to the therapeutic gun is reminiscent of Paul Ehrlich's concepts of the “therapia sterilisans magna” and the “magic bullet,” which emerged around the same time.^43^ In both Ehrlich's and Logan's texts there is a sense of a collective desire for so‐called specifics. The term “specifics” referred to drugs that were said to be able to cure certain diseases.^44^ In Ehrlich and Logan, this desire seems to have materialised in similar metaphors. Yet in Logan's case santonin rematerialised again after he had made an observation on his own body about the release of the active ingredient from a specific kind of tablet, presumably so‐called tabloids from the British company Burroughs Wellcome & Co.:

I took *santonin* and *calomel* tablets made by a well‐known English firm, and found the *santonin* tablets in the chamber next morning exactly as they were swallowed. O the unexpected discoveries in original research! I am now convinced that *santonin* should be used not as bullets, that an ascaris might knock herself insensible against, but as powder, the smell or taste of which will make her sick of her abode.^45^

In addition to guns, Logan's therapeutic arsenal also included tried and tested poison powders.

With the articles by Preston Maxwell and Logan, roundworm infections and santonin therapy in China became the subject of research in the field of so‐called tropical medicine. What can be observed with Maxwell and Logan is the construction and staging of a mechanistic wonder weapon whose functioning is reduced to physical interactions.^46^ In contrast to the colonial miracle, this weapon is precisely predictable and requires professional operation. As specifics, such drugs are universal with respect to the diseases attributed to them. When used for certain diseases, they presumably produce the desired effect in all patients. Unlike chemotherapy and Ehrlich's magic bullet, the focus here was only on the targeted effect and not on the targeted production of drugs with chemical means. Explaining the effect in terms of chemically based mechanisms of action was also not relevant here. Yet, the mechanistic wonder weapon santonin operating in China required a construction process, a construction process that was not limited to China. In this process, the pharmacological properties and effects of santonin described in Europe and North America were incorporated into medical practice.

In China, santonin (re)transformed from a colonial miracle to an allopathic wonder drug, potentially dangerous but capable of working wonders in the unerring medical hand. Despite the supposed complexity of the mechanistic wonder weapon, it was assumed that its obvious effects could impress people across epistemic divides between enlightened Europeans and allegedly superstitious Chinese. In part, the place of the colonial miracle was now filled by the mechanisms of the wonder weapon, whereby it was taken for granted that at least its rough functioning was visible to all. This time it were the Western doctors who initially believed in the mechanistic miracle, not the patients, who in the case of the colonial miracle had been placed in this role by the doctors. The mechanistic wonder weapon also had a colonial aspect. This lay in the fact that the doctors believed that it was obvious to everyone how these drugs worked. Universal epistemic grids were presumed, which were simultaneously universalised with the imperial assemblage. The *coloniality* of the mechanistic wonder weapon lay, among other things, in the fact that the in/between and epistemic differences were negated by setting one's own knowledge as universal. The doctors, who thought they were in control of their rational wonder weapon, believed in miracles themselves, caught up as they were in positivist notions of healing.

## 2. Knowledge on the Therapy of Rare Flukes

In early twentieth‐century China, the *coloniality* of drugs not only materialised in medical practices but also in new pharmaceutical knowledge which was of interest to different imperial agents. This knowledge was often published not by research institutions or recognised parasitologists, but by medical missionaries. In addition to roundworms, several other worm diseases known to Western doctors were described in China, especially southern China, in the early years of the twentieth century. Most of them were diagnosed by detecting worm eggs in the stools. Medical missionaries, who had learned about so‐called tropical diseases and parasitological techniques during their studies, realised that there were gaps to be filled in China. They were keen to seize this research opportunity, and in 1907 several members of the China Medical Missionary Association formed the Research Committee.^47^ In their first project, the Research Committee asked their colleagues in the numerous missionary hospitals scattered around China to check their patients’ stools for worm eggs. In this way, they determined infection rates for the roundworm, the hookworm, *Trichocephalus dispar* and several flukes. The general infection rate for the round worm *Ascaris lumbricoides* was put at 75 %. Several of the single reports claimed that over 80 % of the Chinese were infected with worms.^48^ They thus underpinned the discourse on worm‐infested China scientifically.

Worms such as the filaria *Wuchereria bancrofti* described by Patrick Manson, which belongs to the group of roundworms or nematodes, or *Schistosoma japonicum*, which belongs to the group of flukes or trematodes, could only be seen with a microscope. The hookworm, a nematode, is also hard to see with the naked eye. Since the late nineteenth century, hookworm infections had become a problem in the European mining industry and later on colonial plantations. Several plant‐based drugs were used to treat hookworm infection, including thymol, a mixture of eucalyptus oil, chloroform and castor oil, and chenopodium oil. All of these drugs were linked to colonialism, even the essential oil thymol. At the time, thymol was not distilled from thyme, as the name suggests, but from ajwain seeds, which were cultivated in British India.

These drugs were also employed in China, among others by medical missionaries or doctors employed by coal mines. Although it was not the intention of most of these doctors to generate knowledge about anthelmintics, knowledge on the effects and the dosage of these drugs was generated in the course of treatment. The arrangement *doctor‐patient‐anthelmintic‐worm abortion* functioned as a *knowledge generating structure* (*kgs*). With “*kgs*,” I refer to Hans‐Jörg Rheinberger's concept of the experimental system.^49^ In the course of my research on the history of parasitology in southern China, I reformulated it because the research processes were too dispersed to adhere to the notion of the experimental system. While knowledge also emerged through the differential reproduction of particular research arrangements in *kgs*, they were not thoughtfully installed by a relatively homogeneous group of researchers as Rheinberger's experimental systems. Rather the *kgs* seem to have absorbed missionary doctors and other physicians who had not previously been active in the field. Or, as in the case of the knowledge about anthelmintics, the differential reproduction of therapeutic arrangements set up by doctors with the intention of curing patients produced knowledge. Most of the knowledge concerning the dosage and the effects of anthelmintics produced in China did not attract much attention in the discourse of tropical medicine. As research practices these *kgs* were rather inconspicuous, operating in the shadows of the discourse of tropical medicine. However, some knowledge on rather rare worms was discussed and cited by the international community of tropical medicine. Fragments of knowledge on human parasitic flukes specific to East Asia were of particular interest.

Two kinds of these rare parasitic worms, which soon became one kind, were flukes classified as *Fasciolopsis buski* and *Distomum rathouisi*. At the beginning of the twentieth century, parasitology knew only a few specimens of them. There were also isolated reports of the treatment of such worms with thymol, which was considered as a treatment in Patrick Manson's standard work *Tropical Diseases*.^50^ When Francis Wayland Goddard (1877–1958) of the American Baptist Foreign Missionary Society reported *Fasciolopsis buski* in the city of Shaoxing (Zhejiang province) in 1907, the worm had not been seen for 20 years.^51^ Goddard found parasites unknown to him in the stools of two patients. These were identified as the flukes *Fasciolopsis buski* and *Distomum rathouisi* by the Research Committee.^52^

Goddard had linked the complaints of both patients to worms. The first, a 42‐year‐old woman, had been under his care since September 1906. She complained of general weakness, loss of appetite, occasional mild abdominal pain, and fever. Goddard prescribed her a tonic and the care of her husband, who was in Shanghai at the time. She was then treated at a hospital in Shanghai. According to Goddard's account, she was declared a hopeless case and returned to her home in Zhejiang to die. Goddard had received a letter informing him of this and of the patient's state of health. The letter also told him that she had developed swelling in her feet and legs, which led him to suspect hookworm disease. He visited the patient at home, where he gave her a prescription consisting of eucalyptus oil, chloroform, and castor oil to abort the worms and magnesium sulphate as a purgative. Twelve worms identified as *Distomum rathouisi* were expelled during the first abortion. During the second abortion, carried out with thymol, Goddard also obtained several specimens of *Fasciolopsis buski*. This was the last examination, as the patient died shortly afterwards, having improved in the meantime.^53^ A 6‐year‐old boy was Goddard's second patient. He had been brought to the hospital by his parents because of an enlarged and hardened abdomen, occasional diarrhoea and weight loss. With the eucalyptus formula, about 16 *Distomum rathouisi* were excreted. Later the patient also vomited two worms. Interestingly, Goddard equated the illness caused by this worm with the worm 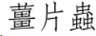 (Jiangpianchong, “ginger slice worm”) known to the local people.^54^

Initially, Goddard seemed to follow the Research Committee's recommendation to classify the worms as *Fasciolopsis buski* and *Distomum rathouisi*. In 1909, however, he claimed to have described his own species, which he christened, at least provisionally, “Shaoxing Fluke.”^55^ Since his 1907 article, he had increasingly examined his patients at the American Baptist Mission Hospital for worm infections. Goddard stated that he had detected worms microscopically in 68 patients, which was 5.5 % of the dispensary's visitors. Of these, an extraordinarily high number, 56 patients, were infected with the Shaoxing Fluke.^56^ In the trematodes, which Goddard initially subdivided into *Fasciolopsis buski* and *Fasciolopsis rathouisi*, he noticed a great variability of forms: “Now I have observed that the flukes ordinarily seen vary considerably in size, becoming shorter and broader if left in water, and seeming to relax and lengthen if dying on a dry surface.”^57^ The form of the flukes here was highly dependent on the time span between the extraction or death of the worms and the examination, as well as on the interventions of the observer.^58^ Given the high prevalence of fluke infection in Shaoxing, Goddard was able to compare large numbers of worms. Hundreds of worms were often found in a single infected person. Observations on the variability of the worms and the realisation that the eggs of the worms showed no differences led Goddard to assume that there was a single species that could not be identified with either *Fasciolopsis buski* or *Fasciolopsis rathouisi*—the “Shaoxing Fluke.”^59^

In the first patient, Goddard had suspected hookworm infection, but flukes were then found on faecal examination. This was preceded by a variation of Goddard's therapeutic practice. It is not clear from the report whether Goddard had previous experience of treating hookworm infection, but he had initially intended to use thymol. As this was not available, he used the eucalyptus oil mixture.^60^ Consequently, the finding of the efficacy of the eucalyptus oil mixture on *Distomum rathouisi* was based on two random variations of *kgs*. In the *doctor‐patient‐anthelmintic‐worm abortion* series, the anthelmintic was varied first, followed by a variation of the aborted worm. Later, Goddard also used thymol, which, in addition to *Distomum rathouisi*, led to the abortion of worms considered *Fasciolopsis buski* at the time.^61^ There was not necessarily a compelling relationship between the variation of the remedy and the effect. However, Goddard's results were consistent with the later realisation that hookworm anthelmintics were also effective against *Fasciolopsis buski*.

Goddard's report not only contained knowledge on anthelmintics, but also on the Shaoxing Fluke. He attributed a great pathogenicity to it. Among others, he described the symptoms of diarrhoea, wasting, jaundice, anaemia, debility and, in children, protuberant abdomen. As the disease progresses, the skin becomes harsh and dry, diarrhoea and debility worsen, and emaciation, nausea, vomiting, dizziness, a gnawing sensation in the abdomen and dyspnoea may also occur. He suspected that the worm's primary location was in the upper small intestine.^62^

In addition to Goddard, several other medical missionaries independently reported abortions of flukes. Arthur F. Cole of the Church Missionary Society's hospital in Ningbo reported on the treatment of two patients. He had found worms in both of them, which he suspected to be *Fasciolopsis buski* and the cause of their complaints. He initially gave them fern extract, which had no noticeable effect.^63^ However, he reported remarkable results with thymol: “One of the most gratifying features of the case was the effect of thymol in transforming the lad from a water‐logged wreck, unable to walk or do anything more than lie in a dyspnœic condition, into an active member of society.”^64^ In 1921, Cole published the results of worm cures in four more cases of *Fasciolopsis buski* infection, some from an endemic area over 100 miles from Ningbo. Cole had since left Ningbo and parts of his research had been destroyed due to armed conflict.^65^ He now considered the bodies of the worms, which had still been fluid ten years earlier, to be one species. Since Cole could not make a clear distinction between the different species, he assumed that all the worms were *Fasciolopsis buski*.^66^ The symptoms he associated with the worm infection included weakness, debility, anaemia, dyspnoea, diarrhoea, oedema, ascites and coughing up worms. Two of the patients were treated by Cole himself with thymol, betanaphthol, and a variation of the eucalyptus oil mixture during his time at the missionary hospital of the Church Missionary Society in Ningbo. The other two were treated by G. J. Evans (Hangzhou) and Allen Carrington Hutcheson (1882–1974) (Jiaxing), where worm cures were carried out with thymol and the eucalyptus oil mixture. In all cases, sometimes hundreds of worms were expelled and in most cases Cole observed a marked improvement in the symptoms. Based on his own observations, thymol worked best.^67^

A few years later, in 1927, Claude Heman Barlow (1876–1969) published a summary of the treatment of *Fasciolopsis* infection in China, which were now treated as a disease entity under the name fasciolopsiasis. Barlow had worked for some time as Goddard's successor at the American Baptist Mission Hospital in Shaoxing. Remarkably, his account does not only list the anthelmintics mentioned above. On the one hand, Barlow described worm cures by Chinese doctors who used betel nut‐derived medicines to kill worms. He assumed that the clinical picture of fasciolopsiasis had been known to them since ancient times, as had the worm *Fasciolopsis buski*, which they called the ginger slice worm (Jiangpianchong, 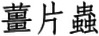). Although Barlow had observed that the dose of betel nut varied from doctor to doctor, he noticed a common pattern in its administration. Betel nut powder was often mixed with a laxative powder and given in bean curd pouches.^68^ That Barlow discussed Chinese medical practices in a scientific text was rather unusual. Most of his colleagues would have ruled this out because of the assumed superiority of the West and racist structures. Barlow was less dismissive towards the Chinese doctors around him. However, even though he was interested in the betel nut therapy, he did not report on its effects and apparently did not test it himself. Barlow assumed that Chinese doctors were now increasingly favouring Western anthelmintics.^69^ Perhaps he did not investigate the efficacy of betel nut further because allopathic doctors in other regions had already evaluated its anthelmintic effect.^70^ Nevertheless, his paper points to the space between Western and Chinese medical practices. Obviously, there was exchange between the doctors. While Barlow reports the adaptation of Western anthelmintics through Chinese doctors, he does not mention the extent to which his conversation with Chinese doctors influenced his own practice.

On the other hand, Barlow described the therapeutic practice of D. Duncan Main. Since the early 1880s, Main had been in charge of the Church Missionary Society's hospital in Hangzhou.^71^ The establishment of the first small hospital in the 1870s had been funded by an anonymous former member of the Indian Civil Service, who wanted to make amends for colonial crimes.^72^ Since the 1890s, Main had administered one to four drachmas of oil of turpentine to worm patients, sometimes in combination with castor oil and chloroform, and had expelled many flukes in this way.^73^ It remains unclear at this point how Main classified the flukes at the time, and whether he regarded these worms and the treatment as new parasitological knowledge.

Main's story provides further clues as to what happened in the in/between. Although the Chinese healers knew more about the ginger slice worm at the time, according to Barlow's account Main was not very interested in their therapies.^74^ Because of the scarcity of the information available, little can be said about the *kgs* that reproduced in Main's practice. Barlow had written to Main to find out about his therapy. Main attempted to present his therapeutic regimen as his own discovery, which obscured the role of *kgs*.^75^ It is likely that Main differentially reproduced instructions based on older dosage instructions for other worms, such as tapeworms. However, it is clear from the account that the *kgs* were in turn reproduced by Chinese healers through his students.^76^ As for the Chinese healers, Barlow assumed that turpentine therapy replaced betel nut treatment.^77^ The in/between could not be controlled by Western doctors. Main's assistants and Chinese healers appropriated the therapy. Barlow rejected their utilisation of turpentine. He claimed that its use often resulted in the side effect of nephritis in their hands.^78^

After testing several anthelmintics for fasciolopsiasis, Barlow himself favoured betanaphthol, a synthetic substance that had been used alongside plant‐based hookworm anthelmintics since 1904. When properly administered, Barlow considered betanaphthol to be not only an effective and inexpensive anthelmintic, but also a safe one that could be given even in “excessive doses.” He referred to a dose of 120 grains that he had taken as part of a self‐experiment. The side effects he mentioned were the pungent taste of the drug, a burning sensation in the digestive tract and a temporary urticaria‐like rash. Barlow recommended administering the highest dose possible to kill a large number of worms at once and to prevent the development of betanaphthol‐resistant worms.^79^ In the light of his experience, he considered dosages such as those recommended for hookworm in Manson's *Tropical Diseases* and used by Cole for infections with *Fasciolopsis buski* to be too low.^80^

The knowledge generated by Goddard, Cole and Barlow on the treatment of *Fasciolopsis* infection, which could be traced back to various *kgs*, became a gap‐filler. Cole explicitly pointed out that such knowledge can only be produced in certain places.^81^ Parasitology was the mainstay of so‐called tropical medicine during these years. When the knowledge about the treatment of flukes such as *Fasciolopsis buski* entered the discourse of tropical medicine, this affected the *coloniality* of the anthelmintics.

## 3. Relationships Between Anthelmintics and Coloniality

Today, the *coloniality* of allopathic medicines materialises when patent‐protected medicines are mainly consumed in the Global North. It is only sporadically that they are distributed from there in a paternalistic manner to the poor in the Global South, who have become dependent on them.^82^ However, medicines are not just something that the Global North has and can generously donate to others, but something in which colonial structures are present and must therefore be the object of decolonisation efforts. Since these medicines are something that we “cannot not want,” decolonisation can only begin by providing equal access to allopathic medicines.^83^ At the same time, however, it is necessary to tackle the colonial structures of drug production. Historical analysis should get involved in this effort.

Anthelmintics were shaped by colonial power relations well before they were used in China. Medicines such as santonin, eucalyptus oil or thymol were means of production for parasitological therapy and knowledge production, and at the same time also partly a production relationship through which colonial exploitation was in some way present in the therapeutic arrangement. The administration of these remedies not only produced therapeutic effects and knowledge about medicines, but also (re)produced power relations. For example, when eucalyptus oil was administered in China to treat hookworm and flukes, it was a remedy that can be traced back to colonial bioprospecting and plantations in Australia or Algeria.^84^ On other colonial plantations, hookworm anthelmintics became *tools of empire* aimed at increasing revenue and the upliftment of colonised societies.^85^ While the *coloniality* of anthelmintics was partly rooted in the harsh exploitation of the colonies, it reproduced in a less drastic manner in China through the staging of anthelmintics for the imperial mission and the production of parasitological knowledge and mechanistic wonder weapons via *kgs*, which had the potential to facilitate further imperialist penetration or colonisation. The role of anthelmintics such as santonin and thymol as *tools of empire* should not be underestimated, even if they do not come close to Daniel Headrick's prime example of quinine.

The use of santonin in the hands of (medical) missionaries was characterised by a reciprocal transformation from the colonial miracle to the mechanistic wonder weapon. Because santonin produced the expected effects, it could be deliberately staged as a colonial miracle and used as a tool in everyday missionary life as the missionaries penetrated into the interior of China. The re‐materialisation of santonin as a mechanistic wonder weapon at the turn of the century led to a change in the way medical practice was conducted, for example, by making patients aware of the adverse effects of therapy and clarifying contra‐indications. For some doctors in particular, this led to a revaluation of drug therapy, which was linked to a collective desire for the mechanistic wonder weapon. One aspect of the *coloniality* of the mechanistic wonder weapon could be seen in the fact that it was inscribed with the conviction that its universal mode of action was easily accessible to all. The mechanistic wonder weapon was not the product of pure reason, it was born between the rational and the irrational. In their use of santonin, (medical) missionaries were constantly moving between the two poles of the colonial miracle and the mechanistic wonder weapon. When the stage was set in the direction of the mechanistic wonder weapon, the colonial miracle became blurred, while other aspects of *coloniality*, such as the production of tropical medical knowledge, became more apparent.

The production of knowledge on anthelmintic therapy exhibited traits of *coloniality* that went beyond the missionary hospitals. In China, *kgs* were reproduced that had developed in the context of hookworm disease in the inner‐European peripheries of coal mines and on colonial plantations. These *kgs* often reproduced not in the context of a research plan, but in therapeutic arrangements. Since therapeutic arrangements always have the potential to generate knowledge, they can be understood as very far‐reaching *kgs*. When therapeutic series reproduced differentially, fragments of knowledge concerning the indications of anthelmintics and dosage regimes emerged and their therapeutic profile was sharpened. Imperialism, and not only Western imperialism, was able to make use of this knowledge. It provided various imperial agents with a sense of security in the space assigned to the “(sub)tropics.” These processes often took place in silence. It is precisely for this reason that *kgs* are relevant to understanding the workings of imperialism. In some cases, therapeutic *kgs* were explicitly placed in a research context, for example as part of the Research Committee, where data on hookworm anthelmintics were statistically analysed.

Some of the knowledge fragments that emerged from the therapeutic series or *kgs* attracted more attention in the discourse of tropical medicine than others. This was particularly true of the knowledge about the flukes described in East Asia, which was partly derived from hookworm *kgs* and was associated with prestige for the researching physicians. The example of Goddard, in particular, shows that *kgs* were again crucial. Goddard found the effectiveness of the eucalyptus mixture while treating hookworm. Rather than being the result of Goddard's plan, operating *kgs* absorbed the alleged author. Like Goddard, many other medical missionaries involved in parasitological research did not come to China to realise research plans. Instead, they took a place in a structure that had already begun elsewhere. Because flukes like *Fasciolopsis buski* were not ubiquitous, the missionary doctors were able to use their findings to fill in gaps in the grid of tropical medicine. This also fuelled the fantasy of being able to control distant regions through complete knowledge, as effective fluke remedies facilitated further expansion efforts. This points to their functioning as a *tool of empire*, which, however, by no means determined these anthelmintics.

The *kgs* involved in parasitological research strongly suggest that these anthelmintics were not the product of a closed European episteme. Sean Hsiang‐Lin Lei has described how Changshan ( 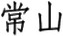, *Diachroa febrifuga* root) emerged from the space between “traditional” Chinese knowledge and “modern” Western knowledge as a biomedical antimalarial drug.^86^ Many other drugs of Western *materia medica* also emerged in part from the interstices between epistemes, when doctors and naturalists appropriated the knowledge of non‐Western healers or when drug production exploited the others of modern man in colonial relations. Goddard's account of the ginger slice worm shows that encounters of the in/between were also involved in the parasitological knowledge production in China. However, the influence of Chinese healers cannot be measured by the publications of Western physicians and missionaries, which form the source base of this paper. Instead, they highlight the efforts of Western doctors to erase their knowledge and the in/between.

The appropriation of knowledge went hand in hand with practises of overcodification, for example the overcodification of the ginger slice worm. The ginger slice worm points to practices and epistemic grids that were related to texts such as the *Bencao Gangmu*. Yet they were distinct assemblages with their own forms and modes of operation. The ginger slice worm differs significantly from the Chinese classics and the nine “worms” (Jiuchong, 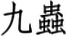) mentioned in them. Its name was not among the Jiuchong. This raises the interesting question for further research as to when and how this worm appeared in Chinese medical practice. Later some authors equated it with the Chichong ( 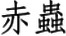), red worm. There may therefore be links to older medical texts. According to the German missionary doctor Gottlieb Olpp (1872–1950), the name ginger slice worm had been used “since ancient times.”^87^ It may well be that the ginger slice worm originated from the developing written knowledge or practices that existed alongside the dominant medical texts.

While medical missionaries made use of the knowledge on the ginger slice worm, they overcoded it as *Fasciolopsis buski* or Shaoxing Fluke, thus negating the in/between and epistemic differences by setting their own knowledge as universal, even by researchers like Goddard who were comparatively open to Chinese practices. Also, the use of Western anthelmintics in everyday practice led to the overcodification of existing knowledge, when the knowledge of Western medicine was presented as universal knowledge and other medical knowledge was relegated to the realm of superstition or traditional knowledge. Overcodification affected not only a single entity like the ginger slice worm, but the wider epistemic grids in which it was embedded. This becomes particularly clear in the case of anthelmintics, as the worms or Chong against which they were used were seen in Chinese societies as particularly changeable beings.^88^ As this affected thinking and being, colonial structures extended into the use of medicines.

## Footnotes

n1 Hickel 2008, on 172–185.

n2 Quijano 2000; Mignolo 2019; Lugones 2010.

n3 For the knowledge of the parasitological worms and their treatment in the period under review, please see Manson 1898; Manson 1914; Mense 1914.

n4 Deleuze and Guattari 2005, e. g. 512–513.

n5 For the history of tropical medicine, see Arnold 1993; Farley 1991; Haynes 2001; Iijima 2010; Li 1999. Some scholars differentiate between a Mansonian tropical medicine, characterised by the paradigm of parasitology, and a pre‐Mansonian tropical medicine. The foundation of the London School of Tropical Medicine in 1899 is often seen as the turning point. Arnold 1996; Worboys 1996.

n6 Ross’ finding that malaria is transmitted by mosquitoes was not only informed by Manson's publications, but he worked very closely with Manson on the matter.

n7 There are several studies on medical missionaries in China. See Andrews 2014; Hardiman 2006; Li 2005; Shemo 2013; Yuan 2021. However, their role in parasitological research has hardly been studied. Gewurtz published on missionaries’ contributions to kala‐azar, a parasitic disease first described in India. Gewurtz 2017. Farley's book *Bilharzia* deals with the history of Claude Heman Barlow (1876–1969), who, as a medical missionary, researched the worm *Fasciolopsis buski* in China. But Farley's focus is on Barlow's Bilharzia work in Egypt after he joined the Rockefeller Foundation's International Health Division, and his research in China is only mentioned in passing. Farley 1991.

n8 See Wahrig and Merdes, “Materialities of the In/between: Drugs and Medical Knowledge between Europe and East Asia,” in this issue.

n9 Headrick 1981.

n10 Li 2005, on 6.

n11 Peake 2014, on 134.

n12 Maxwell undated, on 9.

n13 Kahler 1830b; Alms 1830.

n14 Alms 1831, on 190; Kahler 1830a, on 218.

n15 Flückiger 1867, on 544–545.

n16 Ibid., on 547.

n17 Flückiger 1886, on 2–3.

n18 Ibid., on 1–2.

n19 Ibid., on 3.

n20 Ibid., on 3–4.

n21 Madaus 1938, on 990.

n22 Gilmour 1889b.

n23 Lovett 1895, on 94.

n24 Gilmour 1889a, on 55 and 58.

n25 Gilmour 1889b, on 60.

n26 Ibid.

n27 Ibid., on 61.

n28 It is difficult to tell from Western language sources how Chinese healers, who had their own remedies for roundworms, viewed santonin at the time. According to Gilmour, santonin also met with rejection. Some people had even tried to prove that santonin caused worms, rather than curing worm disease. Ibid.

n29 Ibid., on 62.

n30 Ibid.

n31 Riddel 1891, on 218.

n32 Ibid.

n33 Maxwell 1900, on 56.

n34 Ibid., on 58.

n35 Ibid.

n36 Ibid., on 59.

n37 Ibid., on 57.

n38 Logan 1903, on 149.

n39 Ibid.

n40 Ibid.

n41 Maxwell 1900, on 58.

n42 Logan 1903, on 149.

n43 Jefferys 1908; Jefferys and Maxwell 1910, on 50 and 137; Ehrlich 1909, on 21; Ehrlich 1910, on 229; Prüll et al. 2009.

n44 Maehle 1999, on 285–287.

n45 Logan 1906, on 195.

n46 Merchant 1990.

n47 Research Committee 1907, on 1.

n48 Research Committee 1910.

n49 Rheinberger 1997, on 75–76.

n50 Giles 1890, on 125; Walker 1891; Manson 1898, on 554.

n51 Ward 1909, on 10.

n52 Goddard 1907, on 195.

n53 Ibid., on 196 and 198.

n54 Ibid., on 197.

n55 Goddard 1909.

n56 Ibid., on 387.

n57 Ibid..

n58 Ibid., on 387–388.

n59 Ibid., on 388–389.

n60 Goddard 1907, on 196.

n61 Ibid., on 197.

n62 Goddard 1909, on 388.

n63 Jefferys and Maxwell 1910, on 186.

n64 Ibid.

n65 Cole 1921, on 93.

n66 Ibid., on 93–94.

n67 Ibid., on 94–96.

n68 Barlow 1927, on 253.

n69 Ibid.

n70 Bentley and Trimen 1880, entry 276.

n71 Moule 1897, on 83–84.

n72
*Report* 1884, on 22.

n73 Barlow 1927, on 254.

n74 Ibid.

n75 Ibid., on 253–254

n76 Ibid., on 254.

n77 Ibid., on 264.

n78 Ibid., on 254.

n79 Ibid., on 257–258.

n80 Ibid., on 256.

n81 Cole 1921, on 93.

n82 Although there have been some initiatives at the level of the WTO to address these inequalities, notably the *Doha Declaration on TRIPS and Public Health*, the situation, except maybe from HIV‐medicines, hardly changed. More recently, the debates about vaccine distribution during the COVID‐19 pandemic have shown that the most powerful countries and pharmaceutical companies in the global North are unwilling to give up their privileges.'t Hoen 2019; Thrasher 2024; Wulf 2023.

n83 Spivak 1996, on 28.

n84 Doughty 2000.

n85 Amador 2017; Couacaud 2014.

n86 Lei 2016, on 193–221.

n87 Olpp 1910, on 81.

n88 Nappi 2009, on 106–107.
